# EFCAB10 anchors AK8 to the radial spoke for proper ciliary motility

**DOI:** 10.1073/pnas.2510243122

**Published:** 2025-10-07

**Authors:** Ting Song, Qingchao Li, Qian Lyu, Junkui Zhao, Xirui Zi, Shuxiang Ma, Jiajun Luo, Shushen Li, Shanshan Nai, Hongbin Liu, Xueliang Zhu, Te Li, Jun Zhou, Huijie Zhao

**Affiliations:** ^a^Center for Cell Structure and Function, Shandong Provincial Key Laboratory of Animal Resistance Biology, College of Life Sciences, Shandong Normal University, Jinan 250014, China; ^b^State Key Laboratory of Medicinal Chemical Biology, Haihe Laboratory of Cell Ecosystem, College of Life Sciences, Nankai University, Tianjin 300071, China; ^c^Key Laboratory of Multi-Cell Systems, Shanghai Institute of Biochemistry and Cell Biology, Center for Excellence in Molecular Cell Science, University of Chinese Academy of Sciences, Chinese Academy of Sciences, Shanghai 200031, China; ^d^Cheeloo College of Medicine, Shandong University, Jinan 250012, China

**Keywords:** motile cilia, radial spoke, ciliopathy, EFCAB10, AK8

## Abstract

Radial spokes in motile cilia and flagella regulate rhythmic ciliary motility, which is essential for rapid cell movement and extracellular fluid flow. In humans, defective radial spokes can cause a genetic disorder known as primary ciliary dyskinesia (PCD), resulting in respiratory defects and infertility. Despite their critical role in ciliary motility, the molecular composition and related physiological functions remain to be elucidated. Here, we identify EF-hand calcium-binding domain protein 10 (EFCAB10) and adenylate kinase (AK8) as radial spoke proteins and describe their roles in mouse motile cilia. Using *Efcab10^−/−^* and *Ak8^−/−^* mice, we show that loss of either protein can affect ciliary motility and lead to PCD-related phenotypes in mice. Interestingly, ciliary AK8 is completely absent in *Efcab10^−/−^* cilia, but the loss of AK8 has no effect on ciliary EFCAB10. Further biochemical analyses reveal that EFCAB10 interacts with AK8 and RSPH3B, which fastens AK8 to the radial spoke. Overall, our findings demonstrate the essential role of EFCAB10 as a radial spoke protein in maintaining the integrity of the radial spoke and provide valuable insights into the molecular basis of related ciliopathies.

## Significance

AK8 is an evolutionarily conserved adenylate kinase associated with ciliary motility. However, its precise localization in vertebrate motile cilia remains controversial. Here, we identify an additional radial spoke protein, EFCAB10, that specifically anchors AK8 to the radial spoke. Using *Efcab10* and *Ak8* knockout mouse models, we demonstrate that EFCAB10 deficiency leads to a complete loss of ciliary AK8 in mouse motile cilia, whereas the loss of AK8 has no effect on ciliary EFCAB10. Importantly, the loss of either EFCAB10 or AK8 significantly affects ciliary motility and causes primary ciliary dyskinesia-related phenotypes in mice. Therefore, our findings significantly enhance the understanding of the radial spoke structure and its implications for human diseases.

Motile cilia and flagella are motile organelles present in various unicellular organisms and vertebrates, facilitating cellular locomotion or generating fluid flow over the cell surfaces (1–4). To achieve proper ciliary motility, various supramolecular structures, including dynein arms, radial spokes, and the nexin-dynein regulatory complex, are assembled on the peripheral doublet microtubules, and a central apparatus of two singlet microtubules is formed within the axoneme lumen (1, 5). Mutations in genes involved in the structure or function of motile cilia can lead to a genetic disorder known as primary ciliary dyskinesia (PCD), which affects the respiratory and reproductive systems (3, 6, 7).

The radial spoke complex is a T-shaped structure assembled on the A-tubule of the doublet microtubule with its head toward the central apparatus (1). Pioneering studies of this structure in *Chlamydomonas reinhardtii* have identified many radial spoke proteins and revealed their three-dimensional distribution within the structure (8–10). Although the core structure of the radial spoke is conserved, species-specific differences exist in the molecular composition and interactions within this framework (8, 11). For instance, in *C. reinhardtii*, the only identified flagellar adenylate kinase (AK) is localized to the central apparatus, where it regulates axonemal ATP concentration (12). In contrast, the three cilia-related adenylate kinases in mammals (AK7, AK8, and AK9) have been computationally assigned as radial spoke proteins (13–16). Therefore, it is important to validate their protein–protein interactions and functional roles within this structure.

*Efcab10* encodes an EF-hand calcium-binding domain-containing protein that has been identified as a mouse cilia-related protein (17). Interestingly, a microarray-based study reveals that the expression of *Efcab10* in the mouse lung tissue is controlled by FOXJ1, a key regulator of motile ciliogenesis (18, 19). However, the role of EFCAB10 in motile cilia and its physiological function in vertebrates remain unknown. Here, using *Efcab10* and *Ak8* knockout mice, we demonstrate the essential role of EFCAB10 for anchoring AK8 to the radial spoke, which is vital for proper ciliary motility. Loss of either EFCAB10 or AK8 can lead to PCD-related phenotypes, illustrating their contribution to the pathology of PCD.

## Results and Discussion

### EFCAB10-Deficient Mice Develop PCD-Related Phenotypes.

**Fig. 1.**
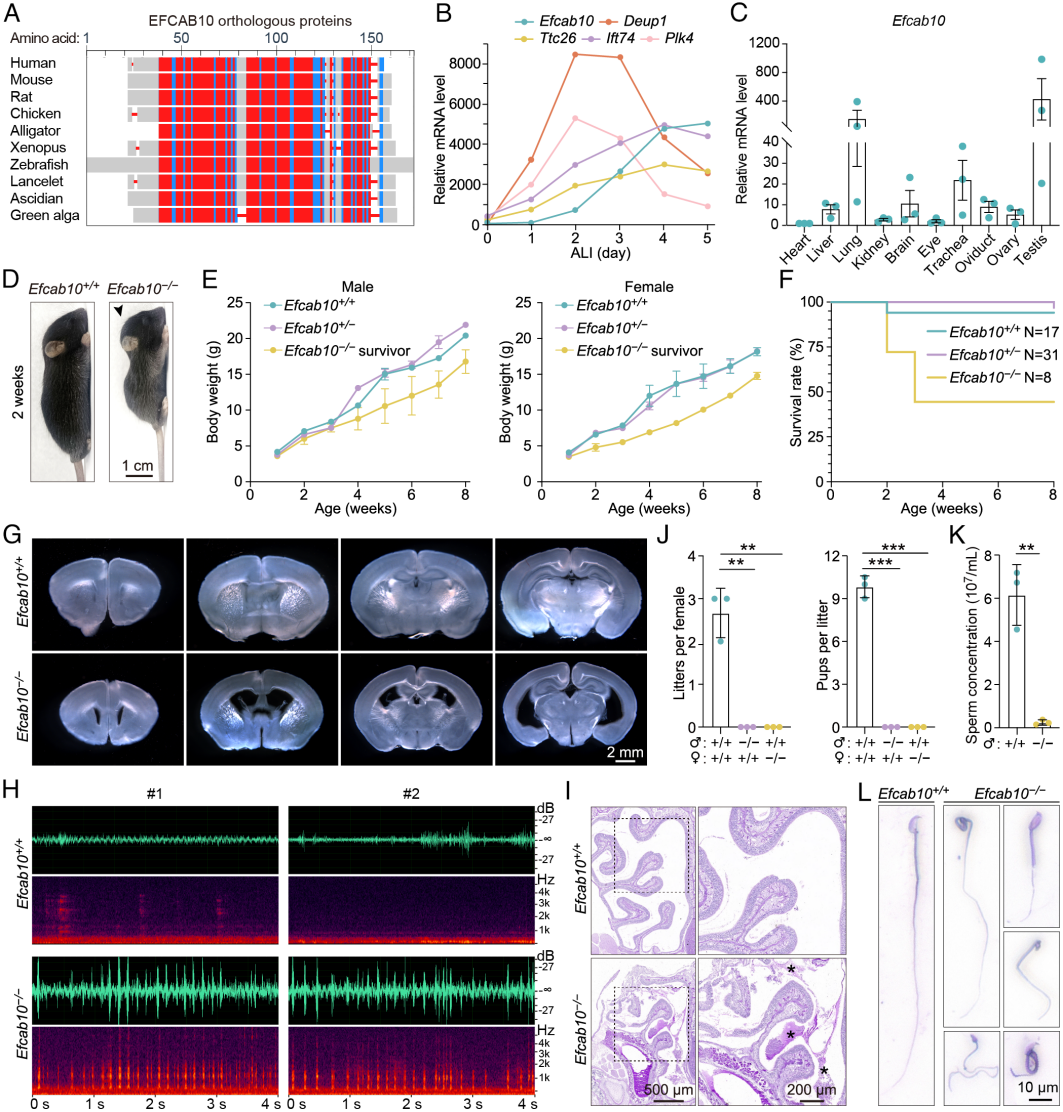
*Efcab10^−/−^* mice display PCD-related phenotypes. (*A*) Multiple sequence alignment of orthologous EFCAB10s with COBALT (Constraint-based multiple alignment tool, NCBI). Highly conserved positions are highlighted in red, and less conserved positions in blue. Protein sequences of orthologous EFCAB10s used are Human (NP_001342455), Mouse (NP_083428), Rat (NP_001102176), Chicken (XP_025009476), Alligator (XP_006026513), *Xenopus* (XP_004913072), Zebrafish (XP_002666930), Lancelet (XP_035676931.1), Ascidian (XP_002131050.1), and Green alga (KAG2450783.1). (*B*) Gene expression profiles of the indicated genes during multiciliogenesis in MTECs. Air–liquid interface (ALI) cultures of MTECs on the indicated day were subjected to microarray analyses. The expression patterns of genes essential for centriole amplification (*Deup1* and *Plk4*) and cilia formation (*Ttc26* and *Ift74*) are included for comparison. (*C*) Real-time PCR analysis showed the expression levels of *Efcab10* in various mouse tissues. *Efcab10* expression was normalized using the corresponding *Gapdh* as the reference gene and baseline 1 (heart) as the reference sample (△△C_T_ method). Data are from three independent biological repeats and presented as mean ± SEM. (*D*) Representative images of 2-wk-old *Efcab10^+/+^* and *Efcab10^−/−^* mice. The *Efcab10^−/−^* mouse was runted with a domed head shape (arrowhead). (*E*) Weights of male and female *Efcab10^+/+^*, *Efcab10^+/−^*, and *Efcab10^−/−^* (survivor) mice were recorded from one to 8 wk of age. (*F*) Survival curves of *Efcab10^+/+^* (starting number: 18), *Efcab10^+/−^* (starting number: 32), and *Efcab10^−/−^* (starting number: 18) mice. The numbers of surviving mice for each genotype at 8 wk of age are shown on the *Right* side. (*G*) Representative images of serial vibratome sections of the brains isolated from 2-wk-old *Efcab10^+/+^* and *Efcab10^−/−^* mice. (*H*) Respiratory sound waveform diagrams of two 4-wk-old *Efcab10^+/+^* and *Efcab10^−/−^* mice. (*I*) PAS staining of the nasal cavities of 2-wk-old *Efcab10^+/+^* and *Efcab10^−/−^* mice. Magnified images of the dashed boxed regions are shown on the *Right*. The asterisks indicate mucus accumulation. (*J*) Fertility assay of *Efcab10^+/+^* and *Efcab10^−/−^* mice in three months. (*K*) Epididymal sperm counts using a hemocytometer. (*L*) Giemsa staining of sperm cells released from the cauda epididymis of 10-wk-old *Efcab10^+/+^* and *Efcab10^−/−^* mice. Data in (*E*, *J*, and *K*) are presented as mean ± SD (n = 3 mice per genotype). Unpaired two-tailed *t* test was performed. **P < 0.01; ***P < 0.001.

We initially identified EFCAB10 in a microarray screen for regulators of multiciliogenesis using mouse tracheal epithelial cells (MTECs) (20). Many hits from this screen are specifically required for different steps of multiciliogenesis, including centriole amplification and cilia formation, with distinct expression patterns during MTEC differentiation (21–26). As an evolutionarily conserved protein, EFCAB10 displayed higher expression levels at late phases of MTEC differentiation (Fig. 1 *A* and *B*). In addition, *Efcab10* mRNA was found to be highly expressed in tissues abundant in motile cilia or flagella, such as the lung, brain, trachea, and testis (Fig. 1*C*). These results suggest its potential role in cilia formation.

To investigate the physiological function of EFCAB10, we generated an *Efcab10* knockout (*Efcab10^−/−^*) mouse model on the C57BL/6J background using CRISPR-Cas9 (*SI Appendix*, Fig. S1*A*). The mouse *Efcab10* gene (Transcript ID: ENSMUST00 000020878.8) contains 4 exons and encodes a protein of 132 amino acids. A pair of guide RNAs (gRNAs) targeting introns 1 and 3 was designed to delete exons 2 and 3 of the mouse *Efcab10* gene, resulting in a 253-bp deletion of the coding region. Founder mice were backcrossed to the C57BL/6J mouse line for two generations to obtain heterozygous mice. The heterozygous animals were viable and indistinguishable from their wild-type littermates. Subsequent matings between F2 (the second filial generation) *Efcab10*^−/−^ mice were used to produce *Efcab10* knockout mice. The offspring genotypes were determined by PCR analysis of genomic DNA from mouse tails (*SI Appendix*, Fig. S1*B*). *Efcab10^−/−^* mice were born at the expected Mendelian ratios (*SI Appendix*, Fig. S1*C*), indicating that EFCAB10 loss does not cause embryonic lethality. Strikingly, we observed that *Efcab10^−/−^* mice grew more slowly and were notably smaller than *Efcab10^+/+^* and *Efcab10^+/−^* littermates, with no sex differences (Fig. 1 *D* and *E*). Meanwhile, *Efcab10^−/−^* mice displayed domed heads at 2 to 3 wk of age, and about 56% of the *Efcab10^−/−^* mice died within 3 wk after birth (Fig. 1 *D* and *F*).

Given that the domed head is a characteristic sign of hydrocephalus, we reasoned that *Efcab10^−/−^* mice may die of lethal hydrocephalus caused by EFCAB10 loss. *Efcab10^+/+^* and *Efcab10^−/−^* brains were thus harvested and analyzed for histological abnormalities. We found that the ventricles of *Efcab10^−/−^* mice were extensively dilated, and the cerebral cortex became thinner compared to *Efcab10^+/+^* mice (Fig. 1*G*). Moreover, the surviving *Efcab10^−/−^* mice were characterized by a hallmark respiratory sound (Fig. 1*H* and Movie S1), indicative of defects in motile cilia that line the airways. Indeed, hematoxylin and eosin staining (H&E) of the paranasal cavity revealed a massive accumulation of protein-rich mucus in *Efcab10^−/−^* mice (Fig. 1*I*). In addition, we examined the fertility of *Efcab10^−/−^* survivors and found that neither sex of *Efcab10^−/−^* mice produced any offspring within the breeding period (three months) (Fig. 1*J*). To explore the fertility defect in *Efcab10^−/−^* males, we evaluated the spermatozoa extracted from the cauda epididymis and found that the counts of spermatozoa in *Efcab10^−/−^* cauda epididymis were significantly reduced, and the *Efcab10^−/−^* spermatozoa exhibited abnormal morphology in the sperm head and flagellum (Fig. 1 *K* and *L*). H&E staining of cauda epididymis sections further confirmed the reduced sperm cells in *Efcab10^−/−^* cauda epididymis (*SI Appendix*, Fig. S1*D*). Furthermore, we conducted H&E staining and immunostaining on testis sections from *Efcab10^+/+^* and *Efcab10^−/−^* mice. In *Efcab10^+/+^* seminiferous tubules, abundant flagellated spermatozoa were observed. In contrast, flagellated spermatozoa were nearly absent within the tubular lumen of *Efcab10^−/−^* testes (*SI Appendix*, Fig. S1 *E* and *F*). Collectively, these results suggest that EFCAB10 deficiency causes PCD-related phenotypes in mice.

### EFCAB10 Deficiency Disrupts the Axonemal Arrangement and Affects Ciliary Motility.

**Fig. 2.**
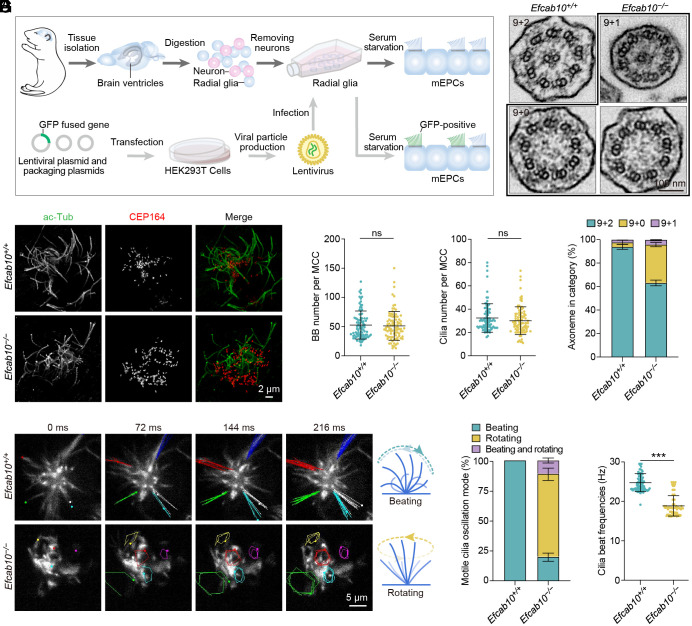
EFCAB10 deficiency affects the axoneme arrangement and ciliary motility. (*A*) Schematic diagrams of lentivirus packaging and infection, and mEPC culture. (*B* and *C*) Immunofluorescence (*B*) and quantifications (*C*) of the number of basal bodies and cilia per multiciliated cell of *Efcab10^+/+^* and *Efcab10^−/−^* mEPCs. Cells were immunostained with acetylated α-tubulin (ace-Tub) and CEP164 antibodies and imaged with 3D-SIM. Ninety cells from three mice (per genotype) were scored using ImageJ. (*D* and *E*) Representative frames (*D*) and quantifications (*E*) of indicated movement modes and beat frequencies of motile cilia in *Efcab10^+/+^* and *Efcab10^−/−^* mEPCs. The trajectories of five cilia in each cell are present. Diagrams illustrate the corresponding ciliary beat patterns. Ninety cells from three mice (per genotype) were scored using ImageJ. (*F* and *G*) TEM images (*F*) and quantifications (*G*) of ciliary axonemes in *Efcab10^+/+^* and *Efcab10^−/−^* mEPCs. At least 134 axonemes from three mice (per genotype) were scored. Data in (*C*, *E*, and *G*) are presented as mean ± SD. Unpaired two-tailed *t* test was performed. ***P < 0.001; ns, not significant.

To determine the effects of EFCAB10 loss on motile cilia, scanning electron microscopy (SEM) was performed to examine the lateral ventricular surface. No apparent morphological differences in motile cilia were observed between *Efcab10^+/+^* and *Efcab10^−/−^* ependyma (*SI Appendix*, Fig. S2). To characterize the cellular causative factors of hydrocephalus in *Efcab10^−/−^* mice, radial glial cells obtained from the brain ependyma were cultured to differentiate into multiciliated mouse ependymal cells (mEPCs) (Fig. 2*A*) (27). Consistent with the SEM results, no significant differences were observed in the numbers of basal bodies or motile cilia between *Efcab10^+/+^* and *Efcab10^−/−^* mEPCs (Fig. 2 *B* and *C*). These data suggest a dispensable role for EFCAB10 in centriole amplification and cilia formation.

Since defective ciliary motility usually causes PCD-related phenotypes (1, 28), we examined ciliary motility in cultured mEPCs. Strikingly, the majority of *Efcab10^−/−^* motile cilia displayed a rotational movement with a significantly decreased frequency, while the *Efcab10^+/+^* motile cilia beat back and forth in a rhythmic manner (Fig. 2 *D* and *E* and Movie S2). Additionally, a group of *Efcab10^−/−^* mEPCs exhibited mixed ciliary movements, featuring both back-and-forth and rotational beating in a single cell (Fig. 2*E*). Furthermore, we examined the ciliary ultrastructures by transmission electron microscopy (TEM). Cross-sections of *Efcab10^+/+^* motile cilia exhibited a normal “9 + 2” axonemal structure, whereas a great portion of *Efcab10^−/−^* motile cilia had deviant axonemal arrangements (Fig. 2 *F* and *G*), including loss of one (“9 + 1”) or both (“9 + 0”) central pair microtubules. Therefore, the flawed ciliary motility in *Efcab10^−/−^* motile cilia might be attributed to the presence of divergent axoneme ultrastructural defects.

### EFCAB10 Anchors AK8 to the Radial Spoke Through Direct Interactions.

**Fig. 3.**
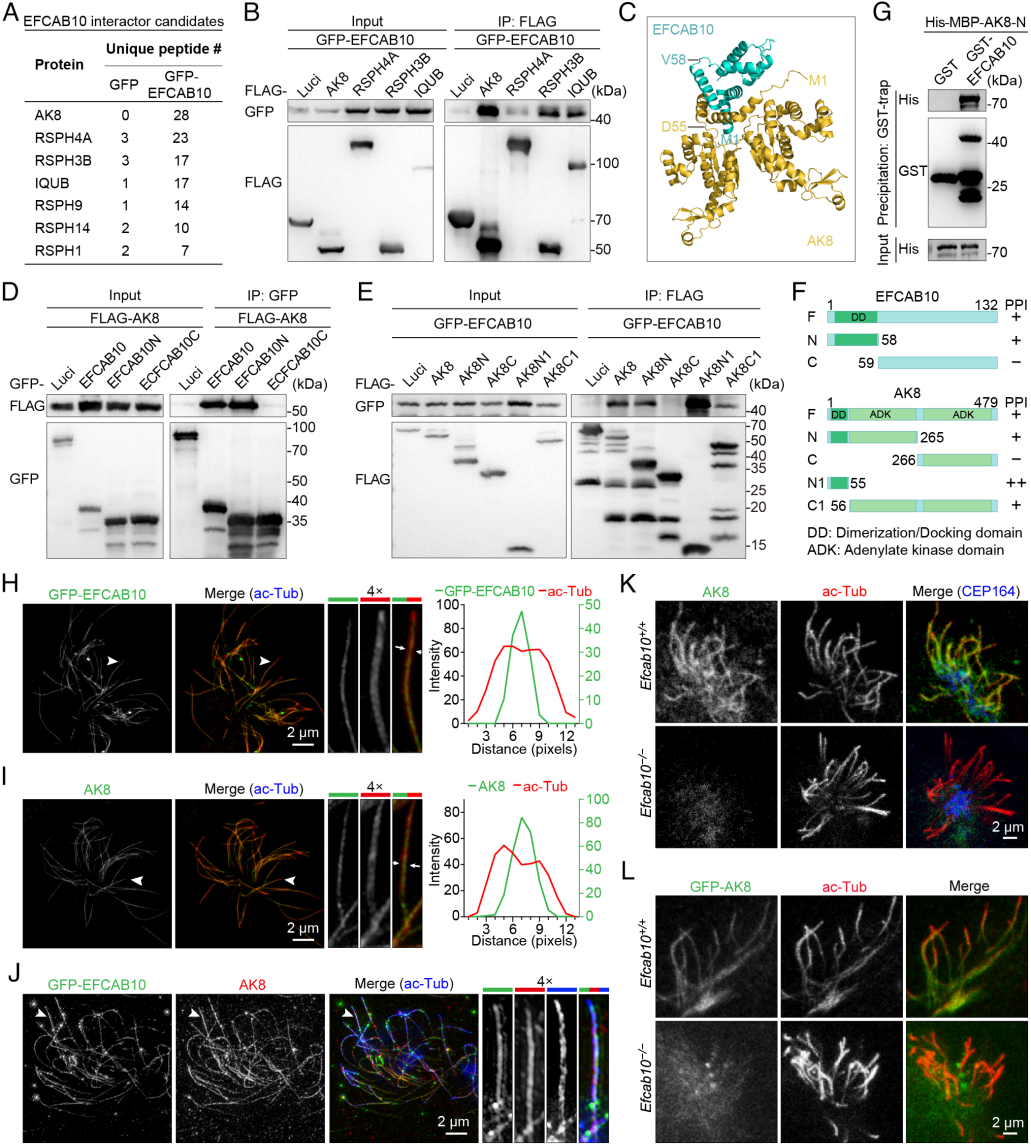
EFCAB10 is essential for anchoring AK8 to the radial spoke. (*A*) EFCAB10 interactor candidates identified by mass spectrometry analysis. (*B*) Coimmunoprecipitation (Co-IP) analysis of coexpressed GFP-EFCAB10 and FLAG-tagged proteins in HEK293T cells. FLAG-tagged proteins were immunoprecipitated with anti-FLAG beads and probed with GFP and FLAG antibodies. Luci, luciferase. (*C*) Binding prediction for EFCAB10 with AK8 by AlphaFold3. Protein chains are colored with PyMOL. The first residue and the amino acid position for generating fragments of each protein are indicated. (*D* and *E*) Co-IP analysis in HEK293T cells exogenously expressing the indicated proteins. GFP-tagged proteins in (*D*) and FLAG-tagged proteins in (*E*) were immunoprecipitated with anti-GFP and anti-FLAG agarose beads, respectively. Blots were probed with the indicated antibodies. Luci, luciferase. (*F*) Schematic diagrams of the truncated fragments of EFCAB10 and AK8 showing the ability to interact. PPI, protein–protein interaction. (*G*) GST pull-down assay using purified GST, GST-tagged EFCAB10, and His-MBP-AK8 fragment proteins. Blots were probed with the indicated antibodies. (*H*–*J*) 3D-SIM images of mEPCs (*I*) or mEPCs expressing GFP-EFCAB10 (*H* and *J*). Cells were immunostained with the indicated antibodies. Magnified images of motile cilia indicated by arrowheads are shown on the right. Line scan graphs show the immunofluorescence intensity along the positions marked by the two arrows in the magnified images. (*K* and *L*) Representative immunofluorescence images of *Efcab10^+/+^* and *Efcab10^−/−^* mEPCs (*K*) or mEPCs expressing GFP-AK8 (*L*). Cells were immunostained with the indicated antibodies.

Next, we used lentivirus carrying the *GFP-Efcab10* fusion gene to infect cultured mEPCs and performed immunoprecipitation followed by mass spectrometry (IP-MS) to identify proteins that interact with EFCAB10 (Fig. 2*A*). Examination of the proteins identified to bind EFCAB10 revealed many components of the radial spoke structure (Fig. 3*A* and *SI Appendix*, Table S1), such as AK8, radial spoke head protein 4 homolog A (RSPH4A), and radial spoke head protein 3 homolog B (RSPH3B) (16, 29–31). To confirm the IP-MS findings and validate the interactions, coimmunoprecipitating complexes were subjected to Western blotting, and results showed that EFCAB10 could be pulled down by all the proteins investigated, with a higher level present in the AK8 pull-down extracts (Fig. 3*B*). We thus further explored the relationship between EFCAB10 and AK8.

The prediction model generated by AlphaFold 3 indicated that EFCAB10 might interact with AK8 via their N-terminus regions (Fig. 3*C*). To evaluate the accuracy of protein interaction prediction, we performed coimmunoprecipitation analysis using various fragments of EFCAB10 and AK8. In agreement with the prediction model, both N-terminus fragment proteins of EFCAB10 and AK8 were found to be sufficient and necessary for their interactions (Fig. 3 *D* and *E*). Interestingly, using the NCBI Conserved Domain Database, we found that both EFCAB10 and AK8 contained an evolutionarily conserved dimerization/docking (DD) domain in their N-terminal regions (Fig. 3*F* and *SI Appendix*, Fig. S3*A*), which mediates their protein–protein interactions. We further performed a GST pulldown assay and found that GST-EFCAB10 could pull down the AK8 N-terminus fragment protein, but not GST (Fig. 3*G*). Collectively, these results indicate that EFCAB10 directly interacts with AK8.

We next examined their cellular localization in multiciliated mEPCs using 3D structured illumination microscopy (3D-SIM). Due to the lack of commercially available antibodies for immunostaining, we generated polyclonal antibodies against EFCAB10 and AK8 and further validated their specificity using knockout animals. However, the EFCAB10 antibody we made remained unsuitable for immunostaining. Therefore, we employed lentivirus-mediated gene transfer to exogenously express GFP-tagged EFCAB10 in mEPCs (Fig. 2*A*). Indeed, both GFP-EFCAB10 and AK8 were located in the ciliary central lumen as defined by acetylated α-tubulin staining (Fig. 3 *H* and *I*). The colocalization of GFP-EFCAB10 and AK8 within the ciliary central lumen was confirmed by 3D-SIM analysis of mEPCs expressing GFP-EFCAB10 (Fig. 3*J*). Notably, upon EFCAB10 depletion, the ciliary signal of AK8 staining was barely detectable, whereas the ciliary localization of other radial spoke components examined was retained, including RSPH1 and RSPH4 (Fig. 3*K* and *SI Appendix*, Fig. S3 *B* and *C*). Moreover, we found that GFP-AK8 also failed to localize to the ciliary axoneme in *Efcab10^−/−^* mEPCs (Fig. 3*L*). Overall, these observations demonstrate that EFCAB10 specifically anchors AK8 to the radial spoke in motile cilia.

### *Ak8*^−/−^ Mice Display a Close Phenocopy of *Efcab10*^−/−^ Mice.

**Fig. 4.**
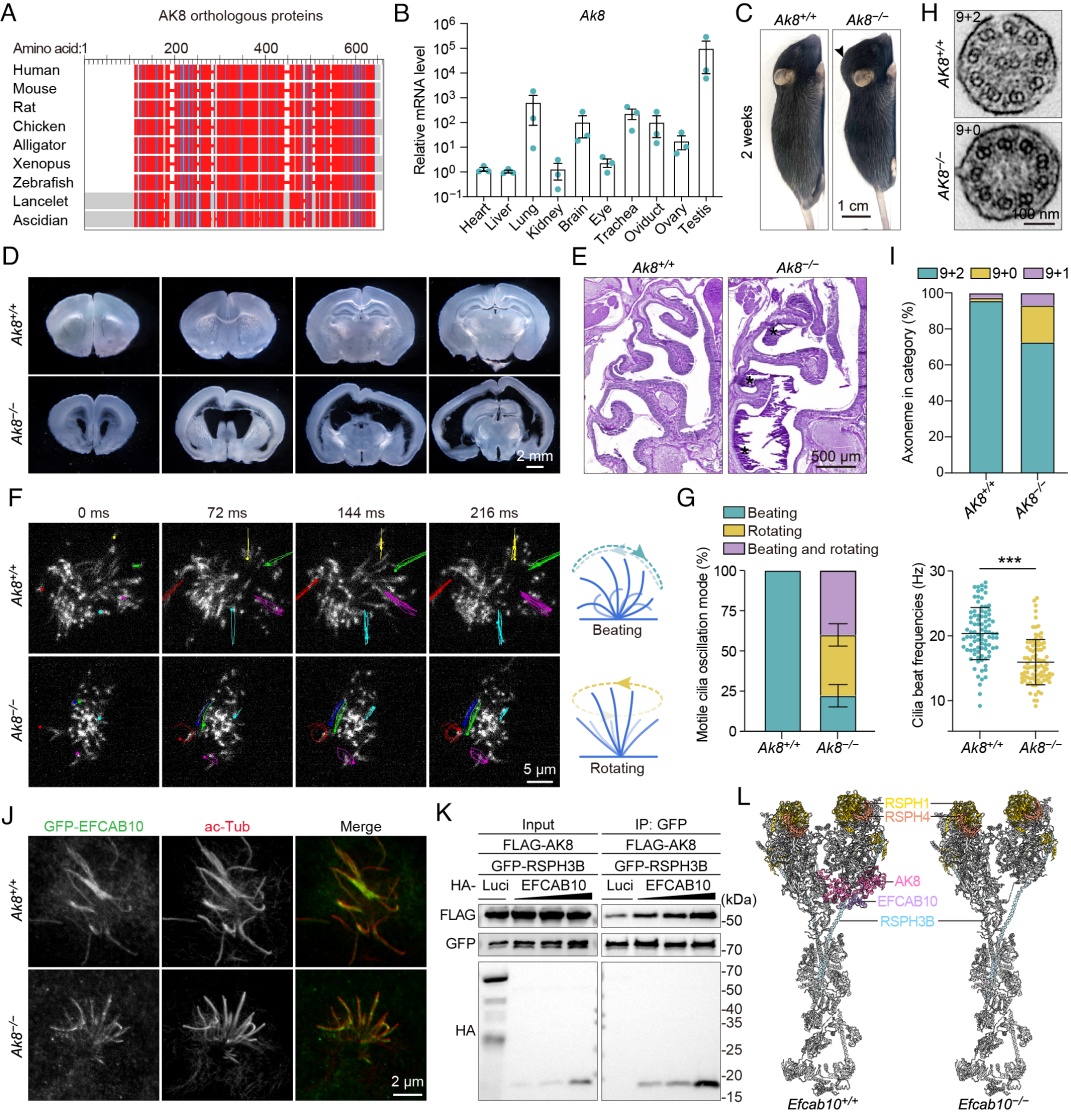
*Ak8* knockout mice display a close phenocopy of *Efcab*10 knockout mice. (*A*) Multiple sequence alignment of orthologous AK8s with COBALT. Highly conserved positions are highlighted in red and less conserved positions in blue. Protein sequences of orthologous AK8s are Human (NP_689785), Mouse (NP_001029046), Rat (NP_001004266), Chicken (XP_428077), Alligator (XP_006029862), *Xenopus* (NP_989104), Zebrafish (NP_001018480), Lancelet (XP_035695553.1), and Ascidian (XP_002124411.1). (*B*) Real-time PCR analysis showed the expression levels of *Ak8* in various mouse tissues. *Ak8* expression was normalized using the corresponding *Gapdh* as the reference gene and baseline 1 (heart) as the reference sample (△△C_T_ method). Data are from three independent biological repeats and presented as mean ± SEM. (*C*) Representative images of 2-wk-old *Ak8^+/+^* and *Ak8^−/−^* mice. The *Ak8^−/−^* mouse exhibits a domed head shape (arrowhead). (*D*) Representative images of serial vibratome sections of the brains isolated from 2-wk-old *Ak8^+/+^* and *Ak8^−/−^* mice. (*E*) PAS staining of the nasal cavities of 2-wk-old *Ak8^+/+^* and *Ak8^−/−^* mice. The asterisks indicate mucus accumulation. (*F* and *G*) Representative frames (*F*) and quantifications (*G*) of indicated movement modes and beat frequencies of motile cilia in *Ak8^+/+^* and *Ak8^−/−^* mEPCs. The trajectories of five cilia in each cell are present. Diagrams illustrate the corresponding ciliary beat patterns. Ninety cells from 3 mice (per genotype) were scored using ImageJ. Data are presented as mean ± SD. Unpaired two-tailed *t* test was performed. ***P < 0.001. (*H* and *I*) TEM images (*H*) and quantifications (*I*) of ciliary axonemes in *Ak8^+/+^* and *Ak8^−/−^* mEPCs. At least 119 axonemes from 2 mice (per genotype) were scored. (*J*) Representative immunofluorescence images of *Ak8^+/+^* and *Ak8^−/−^* mEPCs expressing GFP-EFCAB10. Cells were immunostained with an acetylated α-tubulin (ace-Tub) antibody. (*K*) Co-IP analysis in HEK293T cells exogenously expressing the indicated proteins. GFP-RSPH3B was immunoprecipitated with anti-GFP beads. Blots were probed with the indicated antibodies. Luci, luciferase. (*L*) Proposed atomic model showing the absence of the EFCAB10-AK8 module in the radial spoke structure of *Efcab10^−/−^* mEPCs. This model was generated based on the radial spoke structure of *Bos taurus* (PDB: 9FQR). The predicted protein chains of the indicated proteins are illustrated.

Like EFCAB10, AK8 orthologous proteins are highly conserved across species (Fig. 4*A*). In the mouse, *Ak8* transcripts were also abundantly expressed in certain tissues, such as the testis, the lung, the brain, and the reproductive system (Fig. 4*B*). These results suggest an evolutionarily conserved role for AK8 in motile cilia. However, *Ak8* gene-trap knockout mice on a mixed C57BL and 129 genetic background exhibited normal fertility, with no sinusitis, although these mice did develop late-onset hydrocephalus (15). Since knockout mice on mixed genetic backgrounds usually display a wider range of phenotypes, complicating the assessment of the phenotypes associated with specific genes (32), we generated an *Ak8* knockout (*Ak8^−/−^*) mouse model on the C57BL/6J background using CRISPR/Cas9. The mouse *Ak8* gene (Transcript ID: ENSMUST00000074156.6) contains 13 exons and encodes a protein of 479 amino acids. A pair of guide RNAs (gRNAs) was designed to exons 3 and 4 of the mouse *Ak8* gene. The removal of exons 3 and 4 results in a 164-bp deletion of the coding region and introduces a premature stop codon in exon 5 (*SI Appendix*, Fig. S4*A*). The genome editing was detected by genotyping (*SI Appendix*, Fig. S4*B*). Founder mice were backcrossed to the C57BL/6J mouse line for two generations. The F2 heterozygous animals were crossed to produce *Ak8^−/−^* mice. Moreover, immunostaining of *Ak8^−/−^* mEPCs showed a complete loss of AK8 in motile cilia (*SI Appendix*, Fig. S4*C*), validating the antibody specificity and confirming the absence of AK8 in mutated mice.

Following heterozygous breeding, we found that although *Ak8^−/−^* mice were born at the expected Mendelian ratio, these *Ak8^−/−^* pups displayed growth retardation with evident enlarged dome-shaped heads, and died during the weaning phase (Fig. 4*C* and *SI Appendix*, Fig. S4*D*). Serial sections of the *Ak8^−/−^* brains revealed enlarged ventricles (Fig. 4*D*), indicating severe hydrocephalus in mice with AK8 depletion. However, domed heads and severe postnatal lethality were not observed in *Ak8* gene-trap knockout mice (15), likely due to the mixed genetic background or hypomorphic mutations caused by gene trapping. Moreover, we investigated the respiratory phenotypes in *Ak8^−/−^* mice and observed abundant accumulation of protein-rich mucus in *Ak8^−/−^* sinus cavities (Fig. 4*E*). Together, we conclude that AK8 depletion also results in PCD-related phenotypes in mice, phenocopying the *Efcab10* knockout mice.

Next, we conducted SEM to examine the motile cilia bundles in *Ak8^+/+^* and *Ak8^−/−^* mouse ependyma and found no significant difference in the *Ak8^−/−^* mouse brain (*SI Appendix*, Fig. S4*E*). Consistently, there were no significant differences in the numbers of basal bodies or cilia between *Ak8^+/+^* and *Ak8^−/−^* mEPCs (*SI Appendix*, Fig. S4 *F* and *G*). To determine whether loss of AK8 affects ciliary motility, we performed video microscopy analysis of mEPCs to evaluate the ciliary movement. Upon AK8 depletion, the orderly planar beating pattern was disrupted, with cilia moving rotationally at a lower frequency (Fig. 4 *F* and *G* and Movie S3); similar motility defects were observed in *Efcab10^−/−^* mEPCs (Fig. 2 *D* and *E*). Additionally, a proportion of *Ak8^−/−^* axonemes displayed ultrastructural abnormalities similar to those observed in *Efcab10^−/−^* axonemes (Fig. 4 *H* and *I*). Given the essential role of EFCAB10 in anchoring AK8 to the radial spoke, we investigated whether AK8 depletion may affect the integrity of the radial spoke. Immunostaining with RSPH1 and RSPH4 showed that their ciliary intensity was indistinguishable in *Ak8^−/−^* and *Ak8^+/+^* mEPCs (*SI Appendix*, Fig. S4 *H* and *I*). However, in sharp contrast to the complete loss of ciliary GFP-AK8 in *Efcab10^−/−^* mEPCs (Fig. 3*L*), the ciliary localization of GFP-EFCAB10 in *Ak8^−/−^* mEPCs remained unchanged (Fig. 4*J*). These data further strengthen the conclusion that EFCAB10 specifically tethers AK8 to the radial spoke in motile cilia. Interestingly, we found that the binding of AK8 with RSPH3B was evidently enhanced in the presence of EFCAB10 (Fig. 4*K*). Collectively, these results suggest that EFCAB10 anchors AK8 to the radial spoke by binding to RSPH3B.

In summary, we identify EFCAB10 as an additional radial spoke component that anchors AK8 to the radial spoke through its interactions with RSPH3 and AK8 (Fig. 4*L*). AK8 has been reported to be associated with CFAP45 (33), a protein characterized as a microtubule inner protein (MIP) in the axoneme doublet microtubules (34–37). However, studies employing cryoelectron microscopy and cryoelectron tomography of the vertebrate radial spoke suggest that AK8 is a radial spoke protein, although their proposed locations within this structure differ (16, 30). Interestingly, a recent biochemical study using cross-linking mass spectrometry in *Tetrahymena thermophila* reveals interactions between AK8 and RSPH3 (38). Using knockout mouse models, we clarify that AK8 is anchored to the radial spoke via EFCAB10. The loss of either EFCAB10 or AK8 can affect ciliary motility and cause PCD-related phenotypes in mice. While all the *Ak8^−/−^* mice died before or around weaning time (typically at 3 wk of age), a small portion of the *Efcab10^−/−^* mice were able to survive and reach adulthood, indicating partial compensation for the loss of EFCAB10. Interestingly, male survivors of *Efcab10^−/−^* mice were infertile due to defective spermiogenesis, a phenomenon also observed in *Rsph6a* knockout mice and PCD patients with *RSPH4A* mutations (39, 40). Although there are currently no clinical reports of patients with *EFCAB10* or *AK8* variants, our results provide strong evidence that both EFCAB10 and AK8 are essential components of the radial spoke for proper ciliary motility. As mutations in *EFCAB10* and *AK8* are identified in humans, it will be of interest to determine whether they cause similar defects in axoneme ultrastructure and PCD features. Overall, the findings of this study enhance our understanding of the complex structure and have significant implications for human diseases.

## Materials and Methods

### Plasmid Construction.

The full-length mouse *Efcab10* (NM_029152), *Ak8* (NM_001033874), *Rsph3b* (NM_001083945), *Rsph4a* (NM_001162957), and *Iqub* (NM_172535) were obtained from a mouse cDNA library via PCR. The full-length and corresponding fragment genes were amplified by PCR and subcloned into the recombinant donor vector pDONR221 (Thermo Fisher, 11789100) and then recombined into the Gateway destination vectors (Addgene, 1000000107 and 1000000211) through the LR reaction to generate the indicated expression plasmids. For the construction of prokaryotic expression plasmids, the corresponding full-length or fragment genes were amplified by PCR and subcloned into the pGEX-4 T-1 or pDEST-HisMBP (Addgene, 11085) vectors to generate GST- or HisMBP-tagged constructs. All constructs were validated by Sanger sequencing analysis. Primers used are listed in *SI Appendix*, Table S2.

### Cell Culture.

HEK293T cells (ATCC, CRL-11268) were cultured in Dulbecco’s Modified Eagle’s Medium (DMEM; Thermo Fisher, C11995500BT) supplemented with 10% fetal bovine serum (FBS; Thermo Fisher, A5256701), 1% penicillin/streptomycin (Solarbio, P1400), and 2 mM L-alanyl-L-glutamine (Solarbio, G0190). The HEK293T cell line was routinely tested for *Mycoplasma* contamination. mEPCs were derived from postnatal day 0 (P0) mice. After dissecting the intact brain, the left and right hemispheres were separated, and the meninges, hippocampus, and olfactory bulb were removed. The remaining tissues were digested to single-cell suspensions with papain (Worthington, LS003126) as the digestion enzyme, which were then cultured in DMEM supplemented with 10% FBS, 1% penicillin/streptomycin, 2 mM L-alanyl-l-glutamine, and 1% Primocin (InvivoGen, ant-pm). When reaching 100% confluence, serum was withdrawn from the culture medium for 5 d to induce multiciliogenesis.

### Lentivirus Preparation and Infection.

Lentivirus particles were produced as previously reported (21). In brief, the lentiviral expression plasmid and packaging plasmids (pCMV-Δ8.9 and pCMV-VSVG) were cotransfected into HEK293T cells at a 5:3:2 ratio using the PEI transfection reagent (Polysciences, 23966). The culture medium was changed to fresh complete medium with FBS and antibiotics 8 h after transfection. The culture medium containing lentivirus particles was harvested 48 h after transfection and added to the culture medium of mEPCs at a ratio of 1:20.

### Immunofluorescence Staining and Microscopy.

Cells grown on the cover glasses were fixed with freshly prepared 4% paraformaldehyde (PFA) in PBS for 15 min at room temperature, permeabilized with 0.5% Triton X-100 in PBS for 15 min, and blocked with 4% bovine serum albumin (BSA) in Tris-buffered saline containing 0.1% Tween 20 (TBST) for 1 h. Cell samples were then incubated with primary antibodies and secondary antibodies diluted in the blocking solution overnight at 4 °C and for 1 h at room temperature, respectively. Superresolution images were acquired using a 3D structured illumination microscope (GE Healthcare, Delta Vision OMX SR) at 125 nm intervals and processed with SoftWoRx software. Confocal images were obtained using a Leica TCS SP8 confocal platform equipped with an HCX Plan Apo 63×/1.40 oil immersion objective, with each scanned line averaged four times. Optical sections were captured at 0.5 μm intervals, and z-stack images were processed using maximum intensity projections. All the antibodies used are listed in *SI Appendix*, Table S3.

### Immunoprecipitation and GST Pulldown Assay.

For immunoprecipitation, HEK293T cells expressing the indicated proteins were lysed with a high-salt lysis buffer (1% NP-40, 500 mM NaCl, 50 mM Hepes, 5 mM EDTA, pH 7.8) supplemented with 50 mM NaF, 1 mM Na_3_VO_4_, 3 mM DTT, 1 mM PMSF, and complete protease inhibitors (Calbiochem, 539134). After thorough homogenization, the cell lysate was centrifuged at 14,000×*g* for 10 min at 4 °C. The supernatant was incubated with GFP-Nanoab-Agarose (Lablead, GNA-50-1000) or FLAG-Agarose beads (Sigma, A2220) at 4 °C with gentle rotation for 4 h. The beads were washed with lysis buffer, eluted in sample buffer, and the proteins were analyzed by sodium dodecyl-sulfate polyacrylamide gel electrophoresis (SDS-PAGE).

For the GST-pulldown assay, GST- and HisMBP-tagged proteins were expressed in BL21 CodonPlus (DE3) RIPL bacteria strain (Agilent, 230280). Prokaryotic cells expressing GST-tagged proteins were lysed with a low-salt lysis buffer (20 mM Tris-HCl, 100 mM KCl, 0.1% NP-40, 1 mM EDTA, 10% Glycerol, 10 mM sodium pyrophosphate, pH 7.5) supplemented with 50 mM NaF, 1 mM Na_3_VO_4_, 3 mM DTT, and 1 mM PMSF. GST-tagged proteins were purified with glutathione agarose beads (Sigma, G4510). Prokaryotic cells expressing HisMBP-tagged proteins were lysed with low-salt lysis buffer and centrifuged at 14,000×*g* for 10 min at 4 °C to obtain the supernatant, which was incubated with the GST-tagged protein-bound glutathione agarose beads with gentle rotation for 4 h at 4 °C. The beads were washed again with low-salt lysis buffer and resuspended in 2 × SDS loading buffer for SDS-PAGE analysis.

### Mice.

All the mouse experiments in this study were performed in accordance with the ethical guidelines approved by the Institutional Animal Care and Use Committee of Shandong Normal University (AEECSDNU2024078). The *Efcab10* knockout mouse model (NM-KO-2113123) and *Ak8* knockout mouse model (T031574) were obtained from the Shanghai Model Organisms Center and GemPharmatech, respectively. Both mouse models were generated on the C57BL/6J background. Founder mice of each strain were backcrossed for at least two generations to the C57BL/6J mouse line. The F2 heterozygous animals of each strain were crossed to produce homozygous knockout mice. All knockout mice were genotyped using 2 × Taq Plus Master Mix II (Vazyme, P213). Primers for genotyping are listed in *SI Appendix*, Table S2.

To assess the fertility of mice, three adult (10-wk-old) wild-type and *Efcab10* knockout mice were each continuously paired with an adult wild-type mouse for three months. During the three months, the timing of each litter and the number of pups in each litter were recorded. The pups were removed to allow each mating pair to continue mating until the end of the test. To obtain tissues for RNA extraction, adult mice were killed by decapitation, and tissue samples were dissected and lysed using the TransZol Up Plus RNA kit (TransGen, ER501-01-V2). The RNA was reverse-transcribed to cDNA using the Maxima H Minus cDNA Synthesis Master Mix (Thermo Fisher, M1682). Quantitative real-time PCR was performed using Hieff UNICON advanced qPCR SYBR Master Mix (Yeasen Biotech, 11185ES08), and the results were analyzed with the LightCycler 96 software using the △△C_T_ method. *Gapdh* was used for normalization. For epididymal sperm counting, the cauda epididymis from one side was minced using fine-tipped scissors. Sperm cells were fully released from the epididymis tissues into 1 mL PBS at 37 °C for 30 min. The sperm suspension was then further diluted and counted using a hemocytometer.

### Histological Analysis.

2-wk-old mice were anesthetized with an intraperitoneal injection of 1.25% tribromoethanol (Avertin) at a dose of 250 mg/kg and transcardially perfused with 50 mL PBS and 50 mL of a PBS solution containing 4% PFA. Brains were sectioned into 250-μm-thick sagittal slices and imaged with a Leica M205FA fluorescence stereo microscope. The nasal sinuses were dissected and immediately postfixed in 4% PFA for 24 h. Nasal sinus samples were decalcified overnight in a decalcifying solution, dehydrated using an automatic tissue processor (HistoCore PEARL, Leica), embedded in paraffin, and sectioned at a thickness of 10 mm on a rotary microtome. The sections were laid on the surface of slides, cleaned with xylene, rehydrated, and subsequently stained.

For Periodic Acid–Schiff (PAS) staining, the dewaxed sections were stained with a glycogen PAS staining kit (Nanjing KeyGen Biotech, KGE1103-400) following the manufacturer’s instructions. The stained sections were mounted with neutral resin and thoroughly air-dried in a fume hood. For the Giemsa staining of sperm, sperm from the cauda epididymis of adult wild-type and *Efcab10* knockout mice were released into PBS for 30 min at 37 °C. The sperm suspension was smeared onto adhesive slides, air-dried, and fixed with 4% PFA in PBS for 20 min. The samples were stained with Giemsa staining solution (Beyotime, C0133) following the manufacturer’s instructions and air-dried in a fume hood. Histologically stained sections were imaged using a 3D Histech digital slide scanner (Pannoramic MIDI, 3DHISTECH).

### Electron Microscopy.

Brain and trachea samples were dissected from mice transcardially perfused with 50 mL PBS and 50 mL 4% PFA in PBS. Tissue samples and cultured mEPCs were fixed with a fixative containing 2.5% glutaraldehyde (GA) and 4% PFA at 37 °C for 1 h, followed by fixation overnight at 4 °C. The samples were postfixed in 1% osmium tetroxide for 1 h and then sequentially dehydrated with a graded ethanol series. For SEM, the samples were subjected to critical point drying, coated with gold using the sputtering technique, and examined under a scanning electron microscope (TM3030, Hitachi Asia Ltd) at an accelerating voltage of 15 kV. For TEM, the samples were embedded in Epon 812 resin and polymerized. Ultrathin sections of 60 nm were stained with 2% uranyl acetate for 10 min and 1% lead citrate for 5 min. Images were acquired using an HT-7800 transmission electron microscope (Hitachi Asia Ltd).

### Statistical Analysis.

All experiments were performed with at least three biological repeats. For each experiment, 3 to 4 mice of each genotype were used. For cell immunofluorescence staining, histological staining, and electron microscopy analysis, one representative image from three mice of each genotype was presented. Quantitative results were shown as the mean ± SD unless otherwise specified in the figure legend. Statistical analyses were conducted using GraphPad Prism software. Comparisons between two groups were performed using unpaired two-tailed Student’s *t* tests. Differences were considered statistically significant when the *P* value was less than 0.05.

## Supplementary Material

Appendix 01 (PDF)

Movie S1. Respiratory sound recording of four-week-old *Efcab10^+/+^* and *Efcab10^–/–^* mice.

Movie S2. **Ciliary motilities in representative *Efcab10^+/+^* and *Efcab10^–/–^* mEPCs**. Motilities of multicilia in *Efcab10^+/+^* and *Efcab10^–/–^* mEPCs were stained with SiR-tubulin and live imaged. Image sequences are played back at 5 frames per second.

Movie S3. **Ciliary motilities in representative *Ak8^+/+^* and *Ak8^–/–^* mEPCs**. Motilities of multicilia in Ak8^+/+^ and Ak8^–/–^ KO mEPCs were stained with SiR-tubulin and live imaged. Image sequences are played back at 5 frames per second.

## Data Availability

The data supporting the findings of this study are included in the article and/or supporting information. All software used in this study is commercially available. Materials generated in this study are available from the corresponding author upon reasonable request.
