# Supplementary material for: EFCAB10 anchors AK8 to the radial spoke for proper ciliary motility
Source: Proc Natl Acad Sci U S A. 2025 Oct 7;122(41):e2510243122. doi: 10.1073/pnas.2510243122 (PMC12541429; doi:10.1073/pnas.2510243122)
Supplement: Supplementary file 1 — Appendix 01 (PDF) [file pnas.2510243122.sapp.pdf]

PNAS

Supporting Information for
FCAB10 anchors AK8 to the radial spoke for proper ciliary motility

Ting Song[a,1,2], Qingchao Li[a,1], Qian Lyu[a,1], Junkui Zhao[b,1], Xirui Zi[a], Shuxiang Ma[a], Jiajun Luo[c], Shushen Li[a], Shanshan Nai[a], Hongbin Liu[e], Xueliang Zhu[c,d], Te Li[b], Jun Zhou[a,b,2], and Huijie Zhao[a,2]

Correspondence: 623056@sdnu.edu.cn (T. Song); huijiezhao@sdnu.edu.cn (H. Zhao); junzhou@sdnu.edu.cn (J. Zhou)

This PDF file includes:

Figures S1 to S4
Tables S1 to S3
Legends for Movies S1 to S3

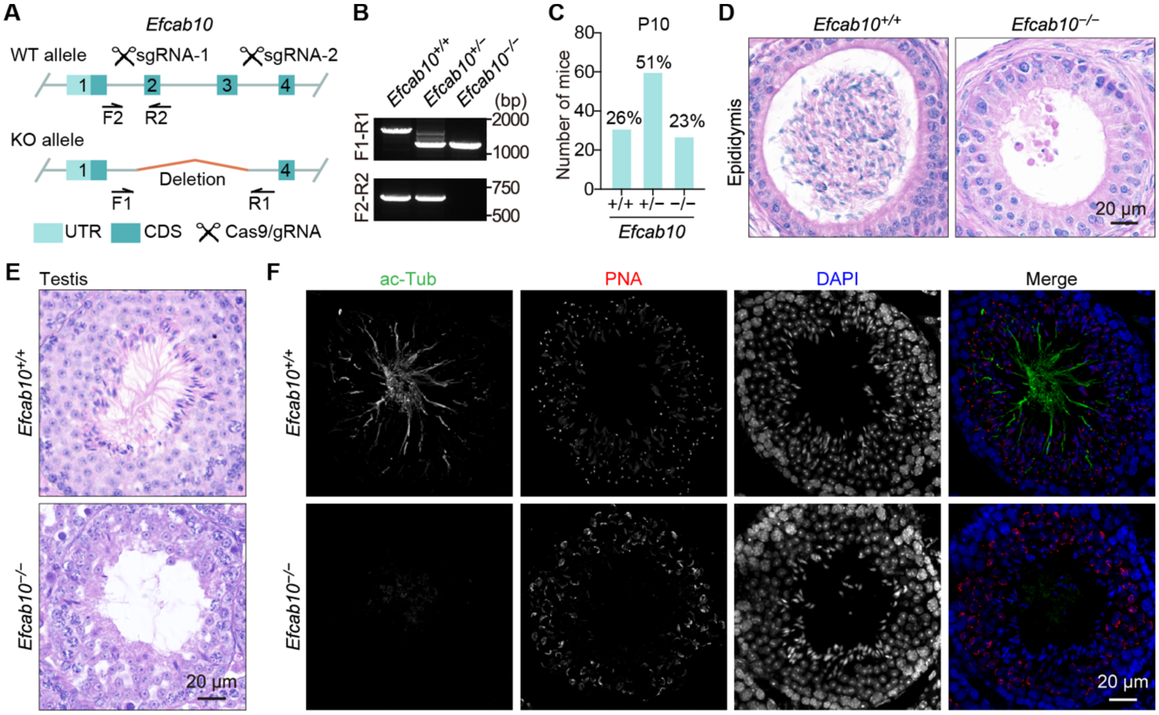

Fig. S1. Generation and acquisition of *Efcab10* knockout mice. (*A*) A schematic diagram of wild-type (WT) and *Efcab10* knockout (KO) alleles. The genomic positions of the primers (F1, R1, F2, and R2) used for genotyping are indicated. UTR, untranslated region; CDS, coding sequence. (*B*) Genotyping of *Efcab10*$^{+/+}$, *Efcab10*$^{+/-}$, and *Efcab10*$^{-/-}$ mice. (*C*) Genotype distribution profile of the offspring from *Efcab10* heterozygous parents at postnatal day 10 (P10) (n = 118 mice). (*D*) H&E staining of *Efcab10*$^{+/+}$ and *Efcab10*$^{-/-}$ epididymis sections. (*E* and *F*) H&E staining (*E*) and immunostaining (*F*) of *Efcab10*$^{+/+}$ and *Efcab10*$^{-/-}$ testis sections. Sections were stained with an acetylated α-tubulin (ace-Tub) antibody, Alexa Fluor 568-conjugated PNA, and DAPI.

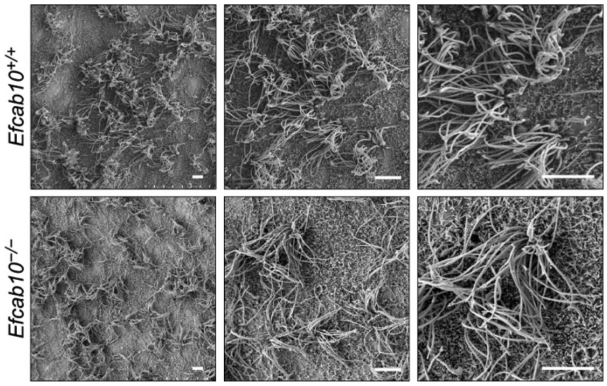

Fig. S2. SEM analysis of ependyma isolated from two-week-old *Efcab10*$^{+/+}$ and *Efcab10*$^{-/-}$ mice. Scale bars, 5 μm.

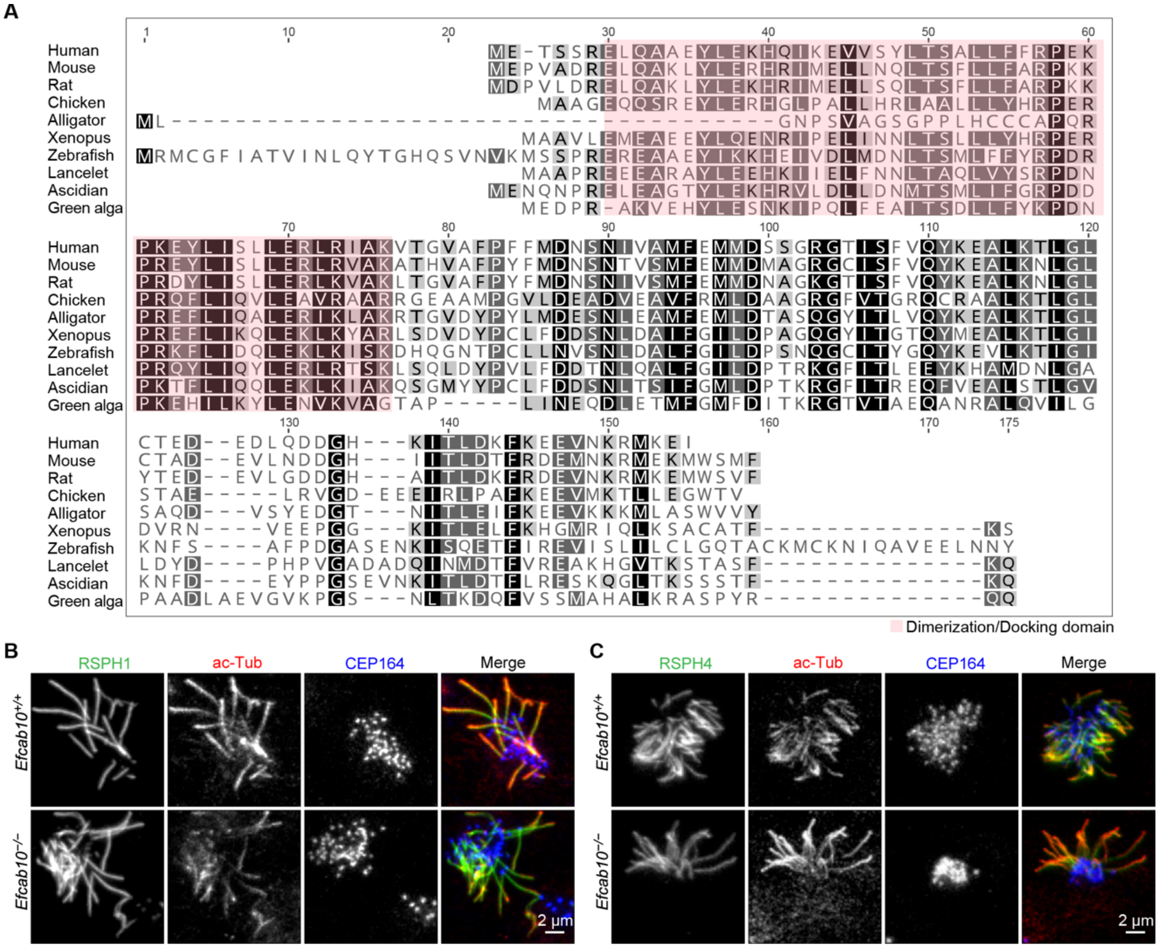

Fig. S3. EFCAB10 is essential for anchoring AK8 to the radial spoke. (*A*) Multiple sequence alignment of orthologous EFCAB10s with the MAFFT program. Fully and strongly conserved residues were highlighted in black and gray, respectively. The conserved dimerization/docking domain of EFCAB10s was highlighted in red. (*B* and *C*) Representative immunofluorescence images of *Efcab10*$^{+/+}$ and *Efcab10*$^{-/-}$ mEPCs immunostained with the indicated antibodies.

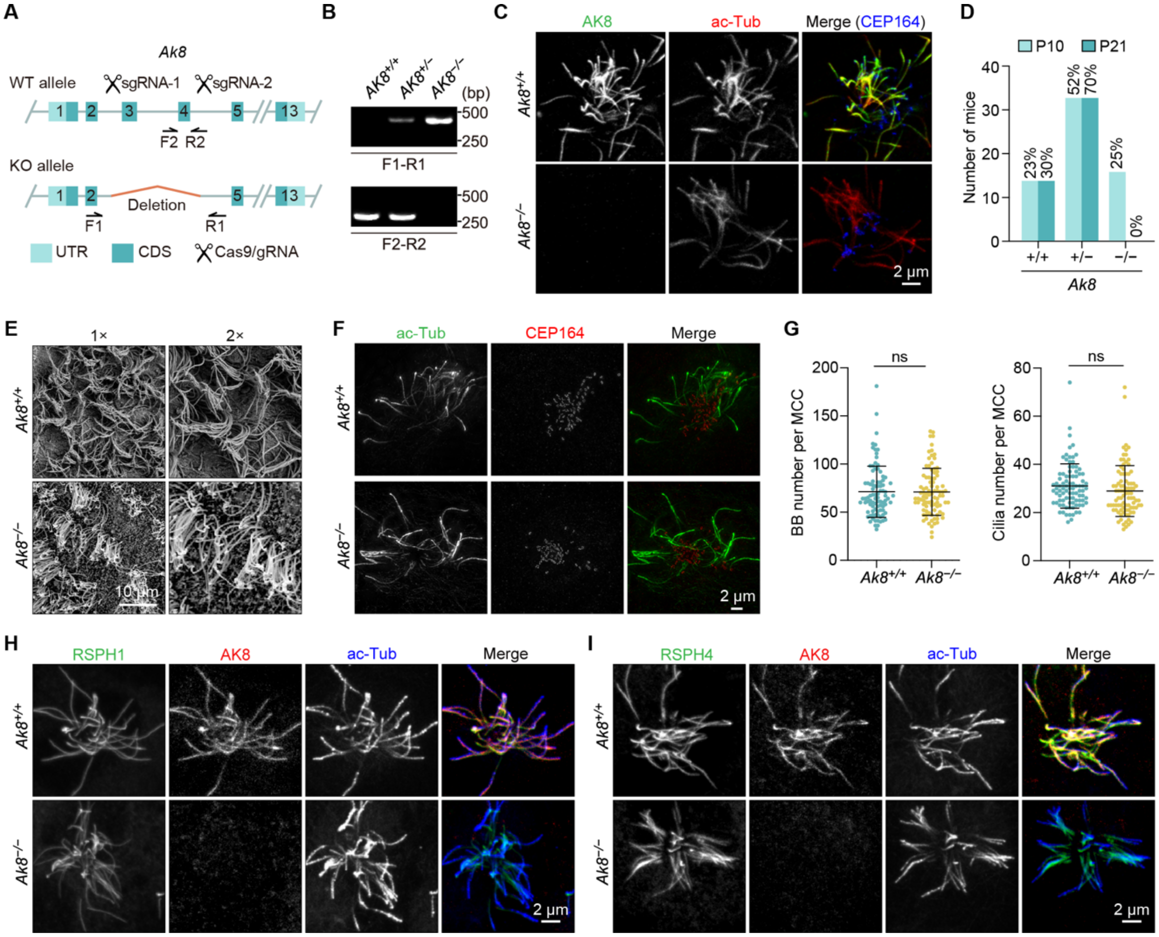

Fig. S4. *Ak8*$^{-/-}$ mice exhibit similar phenotypes to those of *Efcab10*$^{-/-}$ mice. (*A*) A schematic diagram of WT and *Ak8* KO alleles. The genomic positions of the primers (F1, R1, F2, and R2) used for genotyping are indicated. UTR, untranslated region; CDS, coding sequence. (*B*) Genotyping of *Ak8*$^{+/+}$, *Ak8*$^{+/-}$, and *Ak8*$^{-/-}$ mice. (*C*) Representative immunofluorescence images of *Ak8*$^{+/+}$ and *Ak8*$^{-/-}$ mEPCs immunostained with the indicated antibodies. Note that the ciliary labeling of AK8 in *Ak8*$^{-/-}$ mEPCs is invisible. (*D*) Genotype distribution profile of the offspring from *Ak8* heterozygous parents at P10 (n = 63 mice) and P21 (n = 47 mice). (*E*) SEM analysis of ependyma isolated from two-week-old *Ak8*$^{+/+}$ and *Ak8*$^{-/-}$ mice. (*F* and *G*) Immunofluorescence (*F*) and quantifications (*G*) of the number of basal bodies and cilia per multiciliated cell of *Ak8*$^{+/+}$ and *Ak8*$^{-/-}$ mEPCs. Cells were immunostained with acetylated α-tubulin (ace-Tub) and CEP164 antibodies, and imaged with 3D-SIM. 90 cells from 3 mice (per genotype) were scored using ImageJ. Data are presented as mean ± SD. Unpaired two-tailed *t*-test was performed. ns, not significant. (*H* and *I*) Representative immunofluorescence images of *Ak8*$^{+/+}$ and *Ak8*$^{-/-}$ mEPCs. Cells were immunostained with the indicated antibodies.

Table S1. List of EFCAB10 interactor candidates.

Protein	Accession	Description	Unique peptide #	
			GFP	GFP EFCAB10
AK8	Q32M07	Adenylate kinase 8	0	28
RSPH4A	Q8BYM7	Radial spoke head protein 4 homolog A	3	23
RSPH3B	Q9DA80	Radial spoke head protein 3 homolog B	3	17
IQUB	Q8CDK3	IQ and ubiquitin-like domain-containing protein	1	17
RSPH9	Q9D9V4	Radial spoke head protein 9 homolog	1	14
NME5	Q99MH5	Nucleoside diphosphate kinase homolog 5	1	13
DNAJB13	Q80Y75	DnaJ homolog subfamily B member 13	3	11
RSPH14	Q9D3W1	Radial spoke head 14 homolog	2	10
HDAC10	Q6P3E7	Polyamine deacetylase HDAC10	1	9
SPA17	A0A1L1SU87	Sperm surface protein Sp17 (Fragment)	1	8
PPIL6	Q9D6D8	Probable inactive peptidyl-prolyl cis-trans isomerase-like 6	0	8
ROPN1L	Q9EQ00	Ropporin-1-like protein	0	8
RSPH1	A0A494B9F6	Radial spoke head 1 homolog (Chlamydomonas)	2	7
DYDC2	Q9D3X8	DPY30 domain containing 2	0	6
PABPC1	P29341	Polyadenylate-binding protein 1	4	5
DNAJB2	Q9QYI5	DnaJ homolog subfamily B member 2	4	5
CALM3	P0DP28	Calmodulin-3	2	5
EFCAB10	Q9D581	EF-hand calcium-binding domain-containing protein 10	1	5
ARMC2	Q3URY6	Armadillo repeat-containing protein 2	0	5
HDAC6	Q9Z2V5	Histone deacetylase 6	0	5
FHL2	Q543D7	LIM zinc-binding domain-containing protein	4	4
RPS2	Q3TXR5	Small ribosomal subunit protein uS5	4	4
ARF3	Q3U344	ADP-ribosylation factor	4	4
RPS13	Q921R2	Small ribosomal subunit protein uS15	4	4
YBX1	Q71V06	Y box transcription factor (Fragment)	4	4
TUBB4B	P68372	Tubulin beta-4B chain	4	4
HNRNPM	B8JK33	Heterogeneous nuclear ribonucleoprotein M	4	4
HIST2H4	B2RTM0	Histone H4	3	4
CNN3	A0A0G2JDV8	Calponin	3	4
PCBP1	P60335	Poly(rC)-binding protein 1	2	4
PKP1	P97350	Plakophilin-1	2	4
HNRNPU	Q3TS50	B30.2/SPRY domain-containing protein (Fragment)	4	3
RPL27A	P14115	Large ribosomal subunit protein uL15	3	3
RPS16	A4FUS1	Rps16 protein	3	3
MAP1B	B2RQQ5	Microtubule-associated protein 1B	3	3
RPL12	Q8C2K0	Large ribosomal subunit protein uL11	3	3
LMNA	P48678	Prelamin-A/C	3	3

RPS15A	Q5M9M4	Small ribosomal subunit protein uS8	3	3
RPL23	P62830	Large ribosomal subunit protein uL14	3	3
RPL30	A0A2I3BQF4	Large ribosomal subunit protein eL30	3	3
TUBB2A	Q7TMM9	Tubulin beta-2A chain	3	3
RPL24	Q8BP67	Large ribosomal subunit protein eL24	3	3
ASRGL1	Q8C0M9	Isoaspartyl peptidase/L-asparaginase	3	3
PDLIM2	Q8R1G6	PDZ and LIM domain protein 2	3	3
RPL17	Q6PHZ1	Large ribosomal subunit protein uL22	3	3
CRIP2	Q9DCT8	Cysteine-rich protein 2	3	3
RPL38	Q52KP0	Large ribosomal subunit protein eL38	3	3
BAG3	Q9JLV1	BAG family molecular chaperone regulator 3	3	3
AKAP8L	Q3TLG2	C2H2 AKAP95-type domain-containing protein	3	3
HNRNPF	Q9Z2X1	Heterogeneous nuclear ribonucleoprotein F	3	3
CALU	Q3UG11	EF-hand domain-containing protein	2	3
GAPDH	S4R257	Glyceraldehyde-3-phosphate dehydrogenase (Fragment)	2	3
RAN	Q3ULW0	GTP-binding nuclear protein Ran	2	3
ERH	Q4FZH7	Enhancer of rudimentary homolog	2	3
RPLP2	P99027	Large ribosomal subunit protein P2	2	3
ISG15	Q4FJR9	G1p2 protein	2	3
RPL4	Q9D8E6	Large ribosomal subunit protein uL4	2	3
CAT	Q542K4	Catalase	2	3
CAMK4	P08414	Calcium/calmodulin-dependent protein kinase type IV	1	3
HSPA1B	Q3TU85	Hspa1b protein	1	3
RPL3	Q3UB90	60S ribosomal protein L3	1	3
CUL5	E9Q6Z0	Cullin 5	1	3
CNBP	Q3U935	CCHC-type domain-containing protein	1	3
PRDX6	Q6GT24	Peroxiredoxin-6	0	3
CYB5D1	Q5NCY3	Cytochrome b5 domain-containing protein 1	0	3
RBM14	Q3U6A2	RRM domain-containing protein	4	2
FLNC	Q8VHX6	Filamin-C	4	2
RPS6	Q8BT09	40S ribosomal protein S6	3	2
AKAP8	Q059U9	A kinase (PRKA) anchor protein 8	3	2
DNAJB6	A0A0G2JER9	DnaJ heat shock protein family (Hsp40) member B6	2	2
LDHB	D3Z7F0	L-lactate dehydrogenase (Fragment)	2	2
MIF	Q545F0	Macrophage migration inhibitory factor	2	2
RPS20	Q5BLK2	Small ribosomal subunit protein uS10	2	2
HNRNPK	H3BLP7	Heterogeneous nuclear ribonucleoprotein K (Fragment)	2	2
YWHAZ	Q3UA58	14-3-3 domain-containing protein (Fragment)	2	2
RPL19	A2A547	Ribosomal protein L19	2	2
RPS3A1	Q9D1S3	Small ribosomal subunit protein eS1	2	2

RCN1	Q05186	Reticulocalbin-1	2	2
KANK2	Q8BX02	KN motif and ankyrin repeat domain-containing protein 2	2	2
FARSA	E9PWY9	Phenylalanine--tRNA ligase alpha subunit	2	2
DSTN	Q4FK36	Destrin	2	2
HNRNPC	A0A2I3BRM6	Heterogeneous nuclear ribonucleoprotein C (Fragment)	2	2
HNRNPA2B1	O88569	Heterogeneous nuclear ribonucleoproteins A2/B1	1	2
HSP90AA1	Q3TKB9	Histidine kinase/HSP90-like ATPase domain-containing protein (Fragment)	1	2
CFL1	Q544Y7	Cofilin 1, non-muscle	1	2
HNRNPA1	Q3U7F3	RRM domain-containing protein	1	2
PKIA	A0A0A6YXS7	Protein kinase inhibitor, alpha (Fragment)	1	2
ELOC	A0A087WQE6	Elongin-C (Fragment)	1	2
CSRP2	Q6QMT3	CSRP2 (Fragment)	1	2
NPM1	Q5SQB0	Nucleophosmin	1	2
RPS27	A0A0G2JDW7	40S ribosomal protein S27 (Fragment)	1	2
EPPK1	A0A7N9VRC4	Epiplakin 1	1	2
PFN2	D3YWS3	Profilin	1	2
MYH9	Q811J9	Myh9 protein (Fragment)	0	2
UBR4	A2AN08	E3 ubiquitin-protein ligase UBR4	2	1
ZFP326	D3YXQ7	Zinc finger protein 326 (Fragment)	2	1
RPL13A	A0A1B0GSL5	Ribosomal protein L13A	2	1
RPL28	Q5M9N5	Large ribosomal subunit protein eL28	2	1
PPP1CB	P62141	Serine/threonine-protein phosphatase PP1-beta catalytic subunit	2	1
DPYSL3	Q3TAS8	Amidohydrolase-related domain-containing protein	2	1
HNRNPUL2	Q00PI9	Heterogeneous nuclear ribonucleoprotein U-like protein 2	2	1
CIMAP1B	A0A2I3BQ23	Outer dense fiber of sperm tails 3B (Fragment)	2	1
PRDX2	Q61171	Peroxiredoxin-2	2	1
HNRNPAB	Q9D6G1	RRM domain-containing protein	2	1
DHX8	A2A4N9	RNA helicase (Fragment)	1	1
TTLL7	B2RR87	Ttll7 protein	1	1
SPMIP1	B9EJX3	Protein SPMIP1	1	1
HIST1H2AD	B2RVF0	Histone H2A	1	1
CFAP251	E9Q743	Cilia- and flagella-associated protein 251	1	1
PRDX4	B1AZS9	Peroxiredoxin 4 (Fragment)	1	1
RPL21	Q4VA28	60S ribosomal protein L21	1	1
HNRNPH1	Q811L7	Heterogeneous nuclear ribonucleoprotein H1	1	1
SEPTIN7	Q5DTS3	Septin-7 (Fragment)	1	1
KRT1	P04104	Keratin, type II cytoskeletal 1	1	1
ALDOA	D3Z510	fructose-bisphosphate aldolase (Fragment)	1	1
TUBA1B	P05213	Tubulin alpha-1B chain	1	1

TXN	P10639	Thioredoxin	1	1
GSTM1	F6WHQ7	Glutathione S-transferase, mu 1 (Fragment)	1	1
HIST2H2BB	Q8VEE3	Hist2h2bb protein	1	1
RPL7	Q5M9N8	Ribosomal protein L7	1	1
RPSA	A0A1L1SUK3	40S ribosomal protein SA	1	1
LMNB1	Q8C553	IF rod domain-containing protein (Fragment)	1	1
GSTM2	D3YX76	Glutathione S-transferase	1	1
ENO2	A0A0N4SUI6	Enolase 2, gamma neuronal (Fragment)	1	1
LYZ1	A0A077S9N1	Lysozyme f3	1	1
MT3	A0A1D5RLI5	Metallothionein	1	1
SSB	A2AR07	Small RNA binding exonuclease protection factor La (Fragment)	1	1
RPL6	Q3UFI4	60S ribosomal protein L6	1	1
S100A11	P50543	Protein S100-A11	1	1
DSC1	Q8C4K6	Cadherin domain-containing protein	1	1
SNRPA1	G5E883	Small nuclear ribonucleoprotein polypeptide A	1	1
EIF4A1	Q3TSJ4	ATP-dependent RNA helicase	1	1
RPL37A	Q5M9N6	Rpl37a protein	1	1
RPS14	Q3UJS5	40S ribosomal protein S14	1	1
RPS23	Q9CSN9	Small ribosomal subunit protein uS12 (Fragment)	1	1
RPS18	Q561N5	Small ribosomal subunit protein uS13	1	1
RPL18A	A0A1D5RM79	Large ribosomal subunit protein eL20	1	1
RPS15	D3YTQ9	40S ribosomal protein S15	1	1
RPS24	A0A286YEB7	40S ribosomal protein S24	1	1
RPS26	Q497N1	40S ribosomal protein S26	1	1
RPS28	G3UYV7	Small ribosomal subunit protein eS28 (Fragment)	1	1
RPL32	Q5PR09	Ribosomal protein L32	1	1
RPL8	Q9Z237	Ribosomal protein L8 (Fragment)	1	1
HNRNPH2	P70333	Heterogeneous nuclear ribonucleoprotein H2	1	1
FHL1	A2AEY1	Four and a half LIM domains 1 (Fragment)	1	1
TUBB5	P99024	Tubulin beta-5 chain	1	1
GABPB1	A2AQ72	GA repeat binding protein, beta 1 (Fragment)	1	1
FABP5	Q3TLH6	Cytosolic fatty-acid binding proteins domain-containing protein	1	1
COL9A2	Q8K2W0	Collagen, type IX, alpha 2	1	1
KHSRP	A0A3B2W465	KH-type splicing regulatory protein (Fragment)	1	1
XRRA1	A0A140LIG8	X-ray radiation resistance associated 1	1	1
KRT76	Q3UV17	Keratin, type II cytoskeletal 2 oral	1	1
THRAP3	Q8R353	Thrap3 protein	1	1
RNF150	Q5DTZ6	RING finger protein 150	1	1
LUC7L3	F8WGA8	LUC7-like 3 (S. cerevisiae)	1	1
GPRASP1	Q5U4C1	G-protein coupled receptor-associated sorting protein 1	1	1

G0S2	Q545U0	G0/G1 switch protein 2	1	1
H2AC20	Q149V4	Histone H2A	1	1
KAZN	G3UZH4	Kazrin, periplakin interacting protein (Fragment)	1	1
U2AF1L4	E9PWX6	U2 small nuclear RNA auxiliary factor 1-like 4	1	1
MARF1	A0A2R8VH96	Meiosis regulator and mRNA stability 1	1	1
CFAP91	Q8K1N3	Uncharacterized protein	1	1
TMEM200A	A0A1W2P803	Transmembrane protein 200A (Fragment)	1	1
UPP2	A0A2R8W6J9	Uridine phosphorylase	1	1
DDX41	Q3UAC4	RNA helicase	1	1
TRIM34A	Q99PP6	E3 ubiquitin-protein ligase TRIM34A	1	1
FDX2	Q9CPW2	Ferredoxin-2, mitochondrial	1	1
RPL11	Q8VC94	Large ribosomal subunit protein uL5	1	1
RPL34	Q9D1R9	Large ribosomal subunit protein eL34	1	1
S100A14	Q9D2Q8	Protein S100-A14	1	1
TGM1	A0A0R4J293	Transglutaminase 1, K polypeptide	1	1
DNAJA4	D3Z1U5	DnaJ heat shock protein family (Hsp40) member A4 (Fragment)	1	1
SEC11A	D3YWT0	Signal peptidase complex catalytic subunit SEC11	1	1
FBXW13	A0A0G2JFH5	F-box and WD-40 domain protein 13	1	1
SLCO2B1	A0A140LJH7	Solute carrier organic anion transporter family, member 2b1 (Fragment)	1	1
VMN1R76	A0A2I3BQH4	Vomeronasal type-1 receptor	1	1
IGLC2	A0A4E9FSP6	IgL2 (Fragment)	1	1
EFCAB3	B1ASN7	EF-hand calcium binding domain 3 (Fragment)	1	1
PRSS59	D3Z4W5	Protease, serine 59 (Fragment)	1	1
CDK14	E9Q9M2	Cyclin-dependent kinase 14 (Fragment)	1	1
PABPC4L	G5E8X2	Poly(A) binding protein, cytoplasmic 4-like	1	1
HMBOX1	H3BK13	Homeobox containing 1	1	1
PRPSAP2	Q05BD4	Prpsap2 protein	1	1
GM11529	Q3UUH9	Uncharacterized protein	1	1
KRT77	Q4L0E7	Type II cytokeratin Kb39 (Fragment)	1	1
GM5409	Q7M754	Try10-like trypsinogen	1	1
ELMOD2	Q8BHN8	ELMO domain-containing protein	1	1
PRSS3B	Q9D7Y7	Peptidase S1 domain-containing protein	1	1
ACTG1	Q9QZ83	Gamma actin-like protein	1	1
HSPB1	Q9Z2L2	Truncated hsp25 (Fragment)	1	1
GPR179	E9PY61	G-protein coupled receptor 179	0	1
EIF6	A6PWZ2	Eukaryotic translation initiation factor 6 (Fragment)	0	1
MT2	P02798	Metallothionein-2	0	1
ALB	Q546G4	Serum albumin	0	1
LGALS1	P16045	Galectin-1	0	1
HAL	Q9D3I5	Histidine ammonia-lyase	0	1
PSMC2	Q3UIH5	AAA+ ATPase domain-containing protein	0	1

PSMA2	P49722	Proteasome subunit alpha type-2	0	1
RPL10A	Q99JL6	Ribosomal protein	0	1
ATP5F1B	P56480	ATP synthase subunit beta, mitochondrial	0	1
MTPN	P62774	Myotrophin	0	1
DYNLL1	Q9D6F6	Dynein light chain	0	1
CCDC18	Q640L5	Coiled-coil domain-containing protein 18	0	1
SNRNP200	Q7TMG2	Snrnp200 protein (Fragment)	0	1
RPL15	Q5M8Q0	Ribosomal protein L15	0	1
GGCT	A0A0N4SWE8	gamma-glutamylcyclotransferase (Fragment)	0	1
DYDC1	Q9D9T0	DPY30 domain-containing protein 1	0	1
TMED2	Q3THL1	GOLD domain-containing protein	0	1
PSMA1	Q8BTU5	Proteasome subunit alpha type	0	1
MKIAA0705	Q80TU0	MKIAA0705 protein (Fragment)	0	1
HDLBP	A0A087WS92	High density lipoprotein (HDL) binding protein (Fragment)	0	1
RSPH1	Q3YEA1	TSGA2	0	1

Table S2. List of primers used.

Plasmid	Insert	Amino Acids	GenBank	Vector	Sequence (5'->3')
pDONR221-EFCAB10-FL	EFCAB10FL	1-132 aa	NM_029152	pDONR221	GGGGACAAGTTTGTACAAAAAAGCAGGCTTCatggagcctgtggctgac<br>GGGGACCACTTTGTACAAGAAAGCTGGGTCctaaaacattgaccacat
pDONR221-EFCAB10-N	EFCAB10N	1-58 aa	NM_029152	pDONR221	GGGGACAAGTTTGTACAAAAAAGCAGGCTTCatggagcctgtggctgac<br>GGGGACCACTTTGTACAAGAAAGCTGGGTCTTAcacgtgtgttgccttggc
pDONR221-EFCAB10-C	EFCAB10C	59-132 aa	NM_029152	pDONR221	GGGGACAAGTTTGTACAAAAAAGCAGGCTTCgcatttccctactttatg<br>GGGGACCACTTTGTACAAGAAAGCTGGGTCctaaaacattgaccacat
pDONR221-AK8-FL	AK8-FL	1-479 aa	NM_001033874	pDONR221	GGGGACAAGTTTGTACAAAAAAGCAGGCTTCatggatgcaaccacagcccctcat<br>GGGGACCACTTTGTACAAGAAAGCTGGGTCtcaggtaactttcctgggcagagg
pDONR221-AK8-N	AK8-N	1-265 aa	NM_001033874	pDONR221	GGGGACAAGTTTGTACAAAAAAGCAGGCTTCatggatgcaaccacagcccctcat<br>GGGGACCACTTTGTACAAGAAAGCTGGGTCggcattgcatcgatggcc
pDONR221-AK8-C	AK8-C	266-479 aa	NM_001033874	pDONR221	GGGGACAAGTTTGTACAAAAAAGCAGGCTTCccattcacccccaaggtg<br>GGGGACCACTTTGTACAAGAAAGCTGGGTCtcaggtaactttcctgggcagagg
pDONR221-AK8-N1	AK8-N1	1-55 aa	NM_001033874	pDONR221	GGGGACAAGTTTGTACAAAAAAGCAGGCTTCatggatgcaaccacagcccctcat<br>GGGGACCACTTTGTACAAGAAAGCTGGGTCTTAatcattgttcctgcgcag
pDONR221-AK8-C1	AK8-C1	56-479 aa	NM_001033874	pDONR221	GGGGACAAGTTTGTACAAAAAAGCAGGCTTCaatgtgccgaaggttgtg<br>GGGGACCACTTTGTACAAGAAAGCTGGGTCtcaggtaactttcctgggcagagg
pDONR221-RSPH4A	RSPH4A	1-716 aa	NM_001162957	pDONR221	GGGGACAAGTTTGTACAAAAAAGCAGGCTTCatggaaaactctacctctctgaaa<br>GGGGACCACTTTGTACAAGAAAGCTGGGTCttagtcctcatcgtcctcatcttc
pDONR221-RSPH3B	RSPH3B	1-534 aa	NM_001083945	pDONR221	GGGGACAAGTTTGTACAAAAAAGCAGGCTTCatgacagaccgtaaccctcggaca<br>GGGGACCACTTTGTACAAGAAAGCTGGGTCtcactctgccataaggtgtccccc
pDONR221-IQUB	IQUB	1-788 aa	NM_172535	pDONR221	GGGGACAAGTTTGTACAAAAAAGCAGGCTTCatgtctgatcccgaggaagaacgt<br>GGGGACCACTTTGTACAAGAAAGCTGGGTCatgaggttgaatcctacgggttatt
pGEX4T-1-GST-AK8	AK8-FL	1-479 aa	NM_001033874	pGEX4T-1	TCGGATCTGGTTCCGCGTGGATCCatggatgcaaccacagcc<br>CCGCTCGAGTCGACCCGGGAATTCtcaggtaactttcctggg
pET28a-EFCAB10	EFCAB10FL	1-132 aa	NM_029152	pET28a	GGACAGCAAATGGGTCGCGGATCCatggagcctgtggctgac<br>GTGGTGGTGGTGGTGGTGCTCGAGctaaaacattgaccacat
pET28a-AK8-N	AK8-N	1-265 aa	NM_001033874	pET28a	GGACAGCAAATGGGTCGCGGATCCatggatgcaaccacagcc<br>GTGCTCGAGTGCGGCCGCAAGCTTggcattgcatcgatggcc
pLV-GFP-EFCAB10	EFCAB10FL	1-132 aa	NM_029152	pLV-GFP-C1	CCGGACTCAGATCTCGAGCTatggagcctgtggctgac<br>TACCGTCGACTGCAGAATTCctaaaacattgaccacat

	Primers	Sequence (5'->3')
Genotyping	*Efcab10*KO-F1	AGCCTGTTGATCCAGAGCAC
	*Efcab10*KO-R1	ACTGAGCATCCTCCCCTCAT
	*Efcab10*KO-F2	GCATCATGCCAACCCCTTTG
	*Efcab10*KO-R2	CGGCCATGTCCATCATCTCA
	*Ak8*KO-F1	CTAGAAGGGTGTCCATGTGCTTG
	*Ak8*KO-R1	GCTCCTGAAGACTGGCTCTAAATG
	*Ak8*KO-F2	GCTGACCCGGAACTCATTACTAG
	*Ak8*KO-R2	AGATGTTTGCAGAGCCACATG
qPCR	*Efcab10*-F	TCTGTGTACTGCAGATGAAGTTTT
	Efcab10-R	TTCCATCCTCTTGTTCATTTCATC
	Ak8-F	CACAGGAGAAAGGTTCCACC
	Ak8-R	AATGTCTTCAGGGCTTGCTC
	Gapdh-F	AGGTCGGTGTGAACGGATTTG
	Gapdh-R	TGTAGACCATGTAGTTGAGGTCA

Table S3. List of antibodies used.

Primary antibodies					
**Antigen**	**isotype**	**supplier**	**Cat. #**	**WB**	**IF**
Rabbit ant GFP antibody	IgG	ABclonal	AE011	1:20000	
Rabbit anti FLAG antibody	IgG	ABclonal	AE092	1:5000	
Mouse anti HA antibody	IgG1	Abmart	M20003M	1:5000	
Rat anti GFP antibody	IgG2a, k	BioLegend	338002		1:200
Rabbit anti RSPH3B antibody	IgG	Proteintech	17603-1-AP		1:1000
Rabbit anti CEP164 antibody	IgG	Proteintech	22227-1-AP		1:1000
HRP-conjugated His-Tag Antibody	IgG1	Proteintech	HRP-66005	1:5000	
Mouse anti acetylated tubulin antibody	IgG2b	Sigma	T6793		1:1000
Guinea pig anti CEP164 antibody	IgG	home-made			1:1000
Rabbit anti RSPH1 antibody	IgG	home-made			1:1000
Rabbit anti RSPH4A antibody	IgG	home-made			1:1000
Rabbit anti RSPH9 antibody	IgG	home-made			1:1000
Guinea pig anti AK8 antibody	IgG	home-made			1:1000
**Secondary antibodies**					
**Name**	**Label or Dye**	**supplier**	**Cat. #**	**WB**	**IF**
Goat anti-Mouse IgG (H+L)	HRP	Invitrogen	31430	1:20000	
Goat anti-Rabbit IgG (H+L)	HRP	Invitrogen	31460	1:20000	
Donkey anti-Mouse IgG（H+L)	Dylight 405	Jackson ImmunoResearch	715-475-151		1:200
Donkey anti-Guinea pig IgG (H+L)	Alexa Fluor 488	Jackson ImmunoResearch	706-545-148		1:1000
Donkey anti-Guinea pig IgG (H+L)	Alexa Fluor 647	Jackson ImmunoResearch	706-605-148		1:1000
Donkey anti-Rat IgG（H+L)	Alexa Fluor 488	Thermo Fisher	A48269		1:1000
Donkey anti-Rabbit IgG（H+L)	Alexa Fluor 555	Thermo Fisher	A32794		1:1000
Donkey anti-Mouse IgG（H+L)	Alexa Fluor 488	Thermo Fisher	A32766		1:1000
Goat anti-Guinea pig IgG（H+L)	Alexa Fluor 555	Thermo Fisher	A21435		1:1000
Goat Anti-Rat IgG (H+L)	6 nm Colloidal Gold	Jackson ImmunoResearch	112-195-167		1:50
Donkey Anti-Guinea pig IgG (H+L)	6 nm Colloidal Gold	Jackson ImmunoResearch	706-195-148		1:50

Legends for movies

Movie S1 (separate file). Respiratory sound recording of four-week-old *Efcab10*$^{+/+}$ and *Efcab10*$^{-/-}$ mice.

Movie S2 (separate file). Ciliary motilities in representative *Efcab10*$^{+/+}$ and *Efcab10*$^{-/-}$ mEPCs. Motilities of multicilia in *Efcab10*$^{+/+}$ and *Efcab10*$^{-/-}$ mEPCs were stained with SiR-tubulin and live imaged. Image sequences are played back at 5 frames per second.

Movie S3 (separate file). Ciliary motilities in representative *Ak8*$^{+/+}$ and *Ak8*$^{-/-}$ mEPCs. Motilities of multicilia in *Ak8*$^{+/+}$ and *Ak8*$^{-/-}$ KO mEPCs were stained with SiR-tubulin and live imaged. Image sequences are played back at 5 frames per second.
